# Wolcott-Rallison syndrome

**DOI:** 10.1186/1750-1172-5-29

**Published:** 2010-11-04

**Authors:** Cécile Julier, Marc Nicolino

**Affiliations:** 1 Inserm UMR-S 958, Faculté de Médecine Denis-Diderot, Paris, and Centre National de Génotypage, Evry, France; 2 University Paris 7 Denis-Diderot, Paris, France; 3 Hôpital Femme-Mère-Enfant, Division of Pediatric Endocrinology, Lyon University, Lyon, France; 4 INSERM U870, Centre d'Investigation Clinique, Lyon, France

## Abstract

Wolcott-Rallison syndrome (WRS) is a rare autosomal recessive disease, characterized by neonatal/early-onset non-autoimmune insulin-requiring diabetes associated with skeletal dysplasia and growth retardation. Fewer than 60 cases have been described in the literature, although WRS is now recognised as the most frequent cause of neonatal/early-onset diabetes in patients with consanguineous parents. Typically, diabetes occurs before six months of age, and skeletal dysplasia is diagnosed within the first year or two of life. Other manifestations vary between patients in their nature and severity and include frequent episodes of acute liver failure, renal dysfunction, exocrine pancreas insufficiency, intellectual deficit, hypothyroidism, neutropenia and recurrent infections. Bone fractures may be frequent. WRS is caused by mutations in the gene encoding eukaryotic translation initiation factor 2α kinase 3 (EIF2AK3), also known as PKR-like endoplasmic reticulum kinase (PERK). PERK is an endoplasmic reticulum (ER) transmembrane protein, which plays a key role in translation control during the unfolded protein response. ER dysfunction is central to the disease processes. The disease variability appears to be independent of the nature of the *EIF2AK3 *mutations, with the possible exception of an older age at onset; other factors may include other genes, exposure to environmental factors and disease management. WRS should be suspected in any infant who presents with permanent neonatal diabetes associated with skeletal dysplasia and/or episodes of acute liver failure. Molecular genetic testing confirms the diagnosis. Early diagnosis is recommended, in order to ensure rapid intervention for episodes of hepatic failure, which is the most life threatening complication. WRS should be differentiated from other forms of neonatal/early-onset insulin-dependent diabetes based on clinical presentation and genetic testing. Genetic counselling and antenatal diagnosis is recommended for parents of a WRS patient with confirmed *EIF2AK3 *mutation. Close therapeutic monitoring of diabetes and treatment with an insulin pump are recommended because of the risk of acute episodes of hypoglycaemia and ketoacidosis. Interventions under general anaesthesia increase the risk of acute aggravation, because of the toxicity of anaesthetics, and should be avoided. Prognosis is poor and most patients die at a young age. Intervention strategies targeting ER dysfunction provide hope for future therapy and prevention.

## Disease name and synonyms

Wolcott-Rallison syndrome (WRS) was named after Drs Wolcott and Rallison, who first described this syndrome in three affected siblings [1]. It is also known as multiple epiphyseal dysplasia and early-onset diabetes mellitus, according to the main clinical manifestations recognized initially.

## Definition and diagnostic criteria

WRS is characterized by insulin-requiring diabetes that generally appears during the neonatal period or in the first six months of life, with a frequently acute presentation of severe diabetic ketoacidosis at disease onset [2-4]. Two patients with a later onset have been reported, at 14 months [4] and 30 months [2]. Hyperglycemia is initially the only manifestation of the disease, the rest of the biological assessment being normal. Anti-islet cell (ICA), anti-insulin, anti-glutamic acid decarboxylase (GAD) and anti-tyrosine phosphatase (IA2) antibodies are negative. With time, short stature and skeletal dysplasia with radiographic abnormalities progressively develop and are generally diagnosed after diabetes onset, although early signs, including delayed or difficult walking, osteoporosis, deficient mineralization or mild bone abnormalities may be present on close examination and radiography as early as the onset of diabetes. Additional clinical features, which vary between patients, are usually diagnosed after diabetes onset.

## Epidemiology

This is a rare disease, with fewer than 60 cases described in the literature [3,4]. In the large majority of cases, affected individuals are from populations in which consanguineous marriages are frequent, such as the Middle-East, North Africa, Pakistan and Turkey. Despite its recognition as a very rare disease, with very few cases reported in the literature before the first genetic study leading to gene identification, WRS now appears as the most frequent cause of Permanent Neonatal Diabetes Mellitus (PNDM) in consanguineous families ([4] and C. Julier, unpublished data). It is likely that the syndrome was rarely diagnosed before the identification of the responsible gene, and may still be underdiagnosed, as patients may die before expressing the minimum clinical features of neonatal/early-onset diabetes and epiphyseal dysplasia or because the association may not be recognized in younger patients.

## Clinical characteristics

The two main clinical features are well described in the first publication of Wolcott and Rallison [1], and are represented by neonatal/early-onset diabetes and multiple epiphyseal dysplasia. Hepatic dysfunction, manifesting in the form of elevated hepatic enzymes, liver enlargement and recurrent acute liver failure, is the third most frequently observed manifestation, and now appears as a characteristic feature of this syndrome. This review is based on the clinical characteristics of patients in our own experience and on the description of cases reported in the literature, as well as two recent compilations of large series of WRS patients: a description of new cases and literature review of 38 patients from 23 families [3] and a recent study of 29 WRS patients from 25 families [4].

### Diabetes

In the vast majority of cases the onset is observed during the first months of life (extreme ages at onset between 1 day and 30 months). In all but two of the cases reported to date diabetes onset was before the age of 6 months. Diabetes is an obligate feature of WRS, by definition, and it is permanent and totally insulin-dependent from the onset. Although birth weight is generally within normal range, there is now evidence for slightly reduced birth weight in WRS patients (median: -1.4 SD) [4]. The origin of diabetes in WRS is not autoimmune as evidenced by the absence of antibodies specific for type 1 diabetes.

### Bone dysplasia

**Figure 1:**
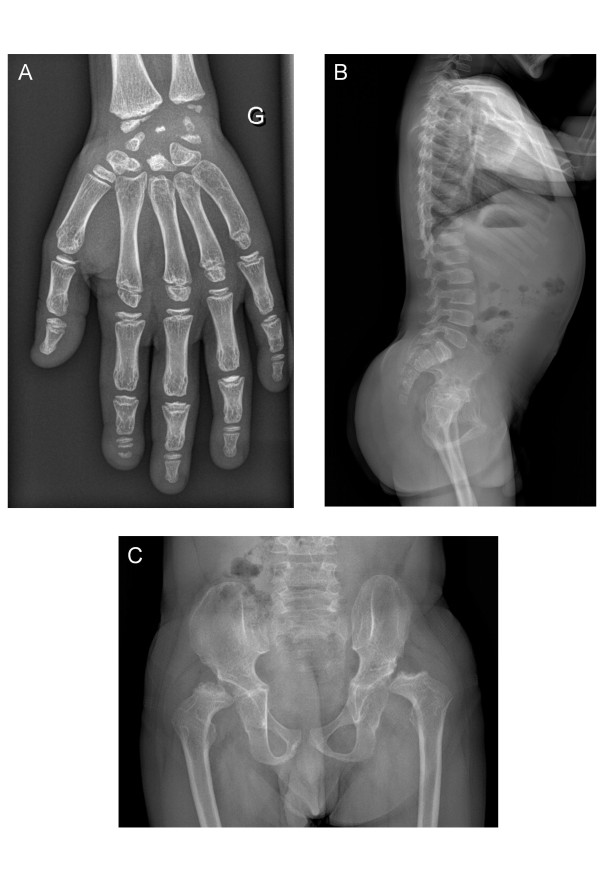
**Radiographs of a WRS patient (age 13 years)**. A. Hand: the carpal bones are small and irregular with dysplastic distal radial and ulnar epiphyses; several phalanges are dysplastic with abnormal metaphyseal cupping at the proximal ends. B. Spine: dorsal region of spine shows flattening of the vertebral bodies with defects at the anterior edges. C. Pelvis: the acetabular roofs are hypoplastic with dysplastic capital femoral epiphyses.

**Figure 2:**
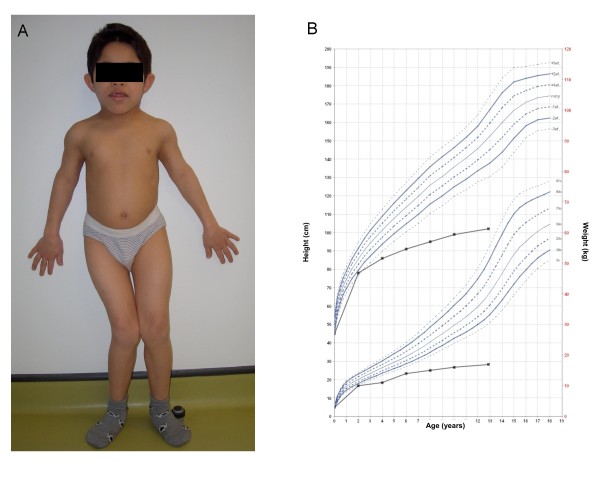
**Skeletal dysplasia and growth retardation in a WRS patient (age 13 years)**. A: Photograph of patient, showing disproportionately short trunk and genu valgum. B: growth chart showing severe dwarfism with poor growth rate.

WRS is characterized by multiple epiphyseo-metaphyseal dysplasia whose major features affect the long bones, pelvis and vertebrae, while the skull is usually normal. Dysplastic changes on knee radiographs are illustrated by enlarged and irregular metaphyses with prominent beaks. Femoral and tibial epiphyses are flattened. On the hand, the carpal bones and phalanges show tubulation defects and appear short and enlarged. The carpal centres are small and irregular, the proximal phalanges show especially dense and cone-shaped epiphyses (Figure 1A) [5]. Interestingly, bone mineralization is affected, as was noted in the early descriptions of the syndrome [6]. Osteopenia is associated with thin cortices and poorly calcified epiphyses which are undeveloped. Multiple and frequent fractures can be observed. Blood calcium and phosphorus values are normal. At the spine, the characteristics correspond to those of mild platyspondyly. Vertebrae show irregular upper and lower plates with frequent ossification defects at the anterior edge (Figure 1B). Changes are especially marked at the dorso-lumbar level with a consequent appearance of thoracic kyphosis and/or lumbar lordosis. Clinically, the chest is rather broad and flattened in the antero-posterior direction (dwarfism with short trunk) (Figure 2A). At the pelvic level, the radiographs usually show abnormal iliac wings and dysplastic acetabular roofs (Figure 1C). Dislocation or subluxation of the femoral heads is a common complication. Femoral epiphyses are flattened with coxa vara. Clinically, difficulty in walking or running is frequent, with a "duck-like" gait related to stiff hips and limited abduction. In some cases, the extreme skeletal condition may manifest with severe cervical spine instability, which may result in spinal cord compression and motor neuropathy in arms and legs (T. Barrett, pers. communication).

### Hepatic dysfunction

Hepatic disease appears to be a characteristic feature of WRS patients. The liver impairment is typically represented by recurrent acute episodes of cytolysis, with or without cholestasis, and even by episodes of liver failure and the possibility of chronic liver dysfunction in older patients. Typically, these episodes are recurrent with spontaneous remission, and their severity is variable. These events are accompanied by an increase in liver volume, which is documented clinically or on ultrasound, with elevated liver enzymes and/or elevated bilirubin levels. They may regress spontaneously or they may be particularly severe, associated with multiorgan failure, and may result in death. These episodes are often triggered by intercurrent diseases such as mild infections of upper airways, and may be complicated by jaundice, hypoglycemia, and even by coma with impaired consciousness in severe cases. Recovery from these events may result in an aggravation of the general condition of patients [7]. Generally, WRS patients have a tendency for recurrent hypoglycemic episodes, most likely as a consequence of liver dysfunction, with impaired gluconeogenesis [8].

### Other clinical features

Other clinical characteristics have been described but are inconsistently present and/or reported. Episodes of impaired renal function, or self-limiting renal insufficiency, are described as occurring concomitantly with hepatic manifestations. Global dysfunction of pancreas is rather rare. In some cases, hypotrophy of pancreas has been documented by ultrasound, computed tomography (CT) or magnetic resonance imagining (MRI) [9,10], and evidence of exocrine pancreatic insufficiency was reported in 8 out of 30 patients in the series of Ozbek et al., four of whom were homozygous for the same mutation [3]. In the series of patients studied by Rubio-Cebezas et al., 2 out of 29 patients required pancreatic enzyme treatment [4]. In terms of neuro-psychological development, intellectual deficit or developmental delay is common, and was reported in 18 out of 29 patients examined [3]. Its severity is variable, and severe cases have been reported, with neuro-motor deficit, microcephaly with simplified gyral pattern and epilepsy [7,11]. Intellectual deterioration is likely to be related in part to secondary brain complications following diabetic coma with ketoacidosis, severe hypoglycaemia, and recurrent episodes of multi-organ failure. In addition, part of the intellectual regression may occur through other mechanisms which are specific to the disease process. Central hypothyroidism has been reported in 6 out of 26 patients in the series of Ozbek et al. [12], who observed that it was only reported during acute episodes, and proposed that central hypothyroidism is not a component of the syndrome, but rather a reflection of euthyroid sick syndrome occurring during severe episodes. Euthyroid sick syndrome is thought to be an energy saving mechanism during stress conditions [13]. Neutropenia has been reported in some patients [6,9], and is often associated with recurring infections. It was reported in 8 out of 21 patients in the series of Ozbek et al. [12]. Other clinical features have been reported in only one or a small number of patients and may be coincidental: discoloration of teeth and skin abnormalities [1], and dysmorphic facies [5,9]. Additional clinical features, including cardiac malformations and pulmonary hypoplasia were also described in a patient diagnosed as WRS, and carrying a mosaic 15q11-12 deletion. However, this case was reported before the genetic elucidation of the syndrome [14], and may correspond to a genetically distinct disease.

## Clinical variability

The clinical course of WRS is very variable, including within the same sibship. Early age at onset of diabetes, bone dysplasia and recurrent hepatic failures, which are the most characteristic features, also show variability between patients. Typically, onset of diabetes occurs before age 6 months and bone dysplasia is diagnosed before one or two years of age. Hepatic episodes may occur at any time during the course of the disease, and may be the first presenting manifestation after diabetes onset. However, age at onset has been reported up to 2.5 years [2] and one patient was described with no bone abnormalities at age 32 years [4]. The presence and severity of other manifestations, i.e. renal failure, exocrine pancreas deficiency, intellectual deficit, hypothyroidism, neutropenia and recurrent infections, are variable between patients. The variability in age at onset, severity and nature of the clinical manifestations and frequency of severe episodes of clinical manifestations explain the variable course of the disease between patients, with survival ranging from a few weeks to 35 years. In some patients, some of the clinical manifestations may be missing due to the young age of the patient, and death may occur before the full diagnosis of WRS is performed.

## Etiology

### Genetics

**Figure 3:**
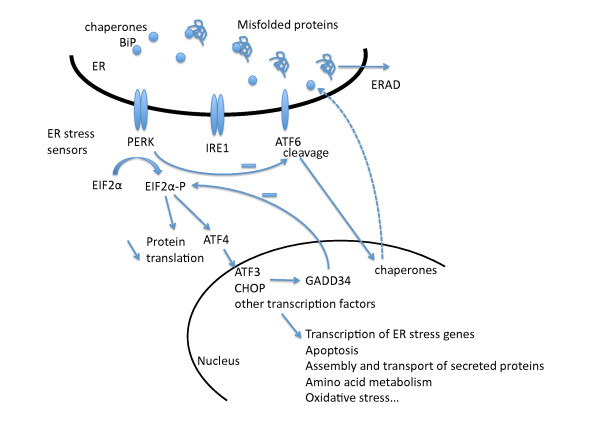
**Schematic representation of the main UPR mechanisms, focusing on PERK related pathways**. Upon accumulation of misfolded proteins in the ER lumen, chaperones such as BiP are displaced from the ER stress sensors PERK, IRE1 and ATF6, resulting in their activation. Upon phospohorylation, PERK assembles in a homodimer (active form), which phosphorylates EIF2α, initiating the downstream UPR response, that reduces the protein overload to the ER: 1) reduction of protein translation and 2) activation of ATF4 and other transcription factors including ATF3 and CHOP, resulting in a variety of cellular and biological effects. ATF3 and CHOP are also involved in a regulatory feedback control of PERK-EIF2α dependent on GADD34. PERK also regulates ATF6, which induces the expression of chaperones, such as BiP and ERp72, which are essentiel in protein processing and quality control. Misfolded proteins are then dissociated by retrotranslocation to the cytosol and degradation by the ubiquitine/proteasome complex (ER associated degradation, or ERAD).

WRS is a rare autosomal recessive disease. Genetic study of two consanguineous families with a total of five patients has established that the disease is caused by mutations in the gene encoding eukaryotic translation initiation factor 2α kinase 3 (EIF2AK3), also known as pancreatic EIF2α kinase (PEK) and PKR-like endoplasmic reticulum kinase (PERK) [15]. In accordance with the general usage, we will denote the gene as *EIF2AK3 *and the protein as PERK. PERK is a transmembrane protein located in the endoplasmic reticulum (ER), which plays a key role in the translation control during the unfolded protein response (UPR). PERK, together with IRE1 and ATF6, is a stress sensor in the ER, which detects the accumulation of misfolded proteins and initiates the appropriate cellular response of UPR that maintains the cell integrity. Upon activation, it phosphorylates the translation initiation factor eIF2α, which in turn reduces protein synthesis, and it also activates the expression of stress-related proteins, such as ATF4, which increases the expression of other transcription factors such as ATF3 and CHOP, that regulate a variety of cellular processes, including amino acid metabolism, oxidative stress, and apoptosis (figure 3). Both mechanisms contribute to preventing overload of the secretory process (for reviews, see [16] and [17]).

**Figure 4:**
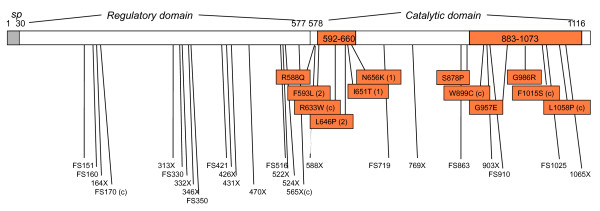
**Schematic representation of PERK and all mutations reported to date**. PERK is composed of a signal peptide (sp), followed by a regulatory domain and a catalytic domain that contains two serine-threonine kinase domains (orange bars). PEK mutations that result in missense mutations are all located within or in the near vicinity of kinase domains (orange blocks). Nonsense (X) and frameshift (FS) mutations, that result in truncated proteins are represented below, and are spread over the length of the protein. Mutations that were found as compound heterozygous in WRS patients are labelled with a "(c)", all others were found in the homozygous state in WRS patients. Mutations homozygous in patients with a relatively late diabetes onset (14 and 30 months) are noted by "(1)" and those homozygous in patients with a relatively longer survival (32 and 35 years) are noted by "(2)". Two mutations were splice mutations located in intron and are not represented. PERK sequence and the position of mutations are provided relative to NCBI RefSeq (NP_004827).

In subsequent studies, many mutations have been reported in the *EIF2AK3 *gene in WRS patients [2-4]. These mutations are either nonsense or frameshift mutations resulting in premature termination of the protein, or missense mutations located in the kinase domains of the protein (Figure 4). Overall, a total of 39 distinct mutations have been reported, 25 (64%) of which are frameshift or nonsense mutations, 12 (31%) missense and 2 (5%) splice mutations. In most families (39/42) these mutations were homozygous, as a consequence of consanguineous or endogamic marriages, and in three cases they were compound heterozygous. To date, all patients presenting "typical" clinical manifestations of WRS have been found to carry homozygous or compound heterozygous mutations in *EIF2AK3*, responsible for the disease. However, in a single patient born from a consanguineous marriage presenting the association of early onset insulin-dependent diabetes (age 18 months) and multiple epiphyseal dysplasia, *EIF2AK3 *mutations and involvement of this gene were excluded [2]. In contrast to all other WRS patients, this patient had none of the other clinical manifestations characteristic of the disease, including episodes of hepatic failure, and had a border-line level of GAD autoantibodies. This may be a variant form of WRS, or it may be a fortuitous association. In another family, a novel mutation in the *PTHR1 *gene was found to be responsible for a form of very rare epiphyseal dysplasia, Eiken syndrome [18], that has been described only in this family [19]; one of the four Eiken patients studied had Type 1 Diabetes (T1D), with juvenile onset and the presence of GAD autoantibodies, which is likely to represent a fortuitous association. It should be noted that syndromic disease presentations occurring in consanguineous families, even in the case of rare diseases, may also reflect coincidental associations.

### Genetic contribution to clinical variability

Remarkably, patients and siblings who share the same mutation in *EIF2AK3 *gene are frequently discordant for their extra-endocrine pancreas manifestations, including intellectual deficit, exocrine pancreas deficiency, hypothyroidism, neutropenia and recurrent infections, and the presence and frequency of episodes of liver failures [2,3]. Therefore the nature of the mutation is not sufficient to explain most of the clinical variability of the syndrome. Neutropenia may be an exception, as it was consistently shared or not shared within patients carrying the same mutation [3]. In particular, it was observed in four out of four patients studied from two Turkish families with the W522X mutation, three out of three French/Tunisian siblings with the Fs346X346 mutation, one child with a Fs910X956 in a single-affected family, and none of the other reported cases [3]. This hypothesis still remains unconfirmed at present, due to the small number of observations, and the apparent absence of any specificity in the nature of the mutation in these cases. In addition, significantly older ages at onset have been reported in two patients, at 30 months and 14 months [2,4]. In the first case, the mutation, N656K, was shown to have a residual kinase activity *in vitro*, which may explain the delayed onset in this patient [2]. Although no activity test was performed for the mutation of the second patient, it is notable that it is located very near the first one, at I650T in the first kinase domain, and we hypothesize that mutations with residual kinase activity may be associated with a delayed age at onset of diabetes, although the rest of the phenotype appears typical of WRS. In addition, a single WRS patient with no skeletal manifestations was reported, who was alive at 32 years old [4]. This patient is homozygous for a F593L mutation. The other patient who survived longer (age 35) was homozygous for a L646P mutation, also located in the first kinase domain. Hence, mutations that have been associated with a relatively milder phenotype (later onset or longer survival) appear to all be missense mutations located in the first kinase domain (Figure 4).

### Pathophysiology

Mice deficient for Perk show a remarkably similar phenotype to human WRS, including permanent neonatal diabetes, growth retardation and skeletal dysplasia, hepatic dysfunction and exocrine pancreas deficiency [20,21]. These mice models have proven very useful to dissect the pathophysiology of WRS. Detailed genetic studies of tissue-specific and cell-specific knockouts showed that diabetes is specifically caused by the loss of Perk expression in β cells [22], exocrine pancreas dysfunction by the loss of Perk expression in acinar cells [23], and skeletal dysplasia and osteopenia by the loss of Perk expression in osteoblasts [24]. These studies showed that Perk is required for fetal and early neonatal development of β cells and for β cell function [22]. As a consequence, Perk knockout (KO) mice have severely reduced β cell mass and insulin secretion, resulting in neonatal diabetes.

In Perk KO mice, hypoglycemia precedes the onset of diabetes, but is absent in pancreas-specific Perk KO [22], suggesting the role of impaired neoglucogenesis due to liver dysfunction. This is likely to contribute to the recurrent hypoglycemia episodes in WRS patients. Although hypoglycemia has not been reported in WRS patients before diabetes onset, to our knowledge, we cannot exclude that such episodes may be undiagnosed, in the absence of other clinical manifestations prior to diabetes onset, and may result in early death of these patients before diagnosis, as observed in Perk KO mice in some specific genetic background (D. Cavener, pers. communication).

In addition, growth retardation in Perk KO mouse has been associated with reduced liver IGF-1 expression during the neonatal period, and could be partly corrected by IGF-1 injections [25], again involving liver dysfunction in this specific feature of WRS.

Initially, in view of the known role of PERK in the UPR, it was proposed that PERK deficiency may result in a defect of the actively secreting β cell to handle the ER stress resulting from this high demand, leading to an accumulation of misfolded proteins, that would become toxic to the cell and lead to apoptosis, resulting in diabetes, independently of an autoimmune process [20,26]. This hypothesis received further support from the study of other monogenic diabetes, where a role of ER stress of the β cell may also be implicated, including Wolfram syndrome (WFS), caused by mutations in the ER Ca2+ channel gene *WFS1 *[27], and neonatal diabetes caused by structural mutations in the insulin gene itself [28]. A role for ER stress has also been proposed as one of the key mechanisms leading to frequent forms of diabetes, including type 1 and type 2 diabetes [17,20,29], as well as a wide range of other common diseases, including neurodegenerative (Huntington's disease, Alzheimer's disease), inflammatory, cardiac and metabolic diseases [30,31].

Recently, however, this hypothesis has been questioned, following detailed studies of β cell development in Perk KO mice performed by D. Cavener's group [22], which showed that these mice had severely impaired β cell proliferation and differentiation during the critical fetal and neonatal periods, resulting in low β cell mass; in addition, these mice have defective proinsulin trafficking, and abrogation of insulin secretion. Further detailed studies performed in β cell lines and in Perk KO mice showed that Perk ablation resulted in reduced cell proliferation and insulin secretion [32]. However, both in Perk deficient mice and cell lines, there was no evidence of uncontrolled protein synthesis and of activation of the ER stress specific pathway and cell death. Rather, there was impaired ER to Golgi trafficking and defective ER associated degradation (ERAD), suggesting that ER dysfunction is responsible for the decreased cell proliferation, abnormal insulin trafficking and insulin secretion observed in β cells of KO mice and WRS patients [32,33].

## Histological features

Histological studies performed on the bone, pancreas and liver of WRS subjects have shown very specific features, that are valuable to our understanding of disease mechanisms. In the first publications describing this syndrome, a defect in ossification was well documented on bone biopsy, with particular abnormalities of the cartilage in the form of hypertrophic chondrocytes [1,34,35]. Further studies reported an irregular proliferation of chondrocytes in the resting cartilage, with the presence of excessively thin ramified collagen fibers in the spongy bone [9].

**Figure 5:**
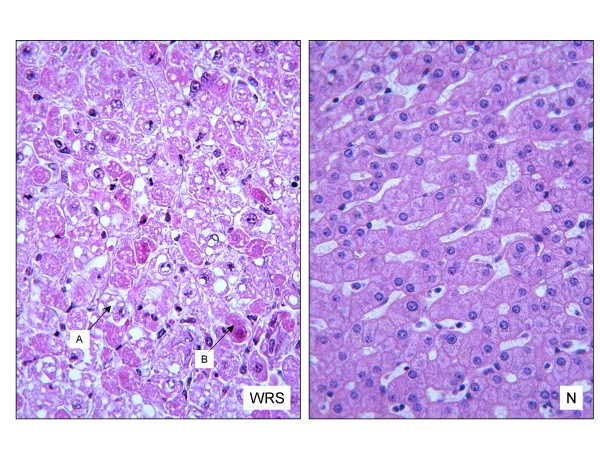
**Liver histology**. Hematoxylin phloxin saffron stain (× 400). Arrows show ballooning aspect of hepatocytes with steatosis (A) and necrosis with accumulation of hyperacidophilic intracytoplasmic material (B). WRS: WRS patient, N: normal control. (histological study performed by R. Bouvier and S. Collardeau-Frachon, Lyon).

In the liver, biopsies performed outside of the recurrent episodes of liver dysfunction typically show progressive fibrosis with mild steatosis and intrahepatic cholestasis [7]. Postmortem examination showed steatosis and lesions of massive necrosis, which is non-inflammatory and has a very remarkable aspect, where cells are isolated or grouped in clusters juxtaposed to normal hepatocytes, producing a mosaic appearance (Figure 5; S. Collardeau-Frachon, pers. communication). Similar necrotic lesions were also observed in the kidney (S. Collardeau-Frachon, pers. communication).

**Figure 6:**
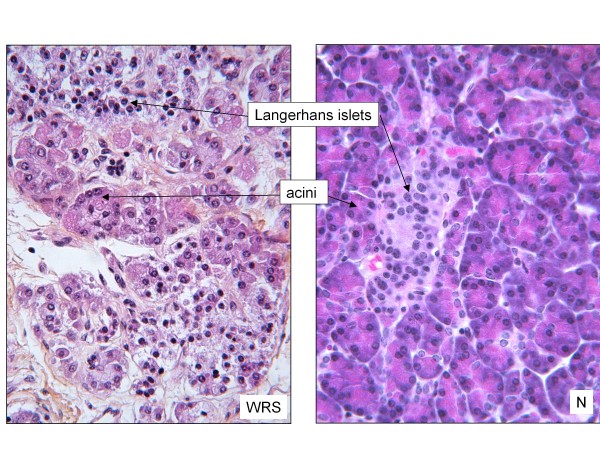
**Pancreatic histology**. Hematoxylin phloxin saffron stain (× 400). Acinar structures are reduced in number and exocrine cells show abundant cytoplasm and hyperacidophilic material in WRS patient. Langerhans islets are smaller in WRS than in normal control. WRS: WRS patient, N: normal control. (histological study performed by R. Bouvier and S. Collardeau-Frachon, Lyon).

**Figure 7:**
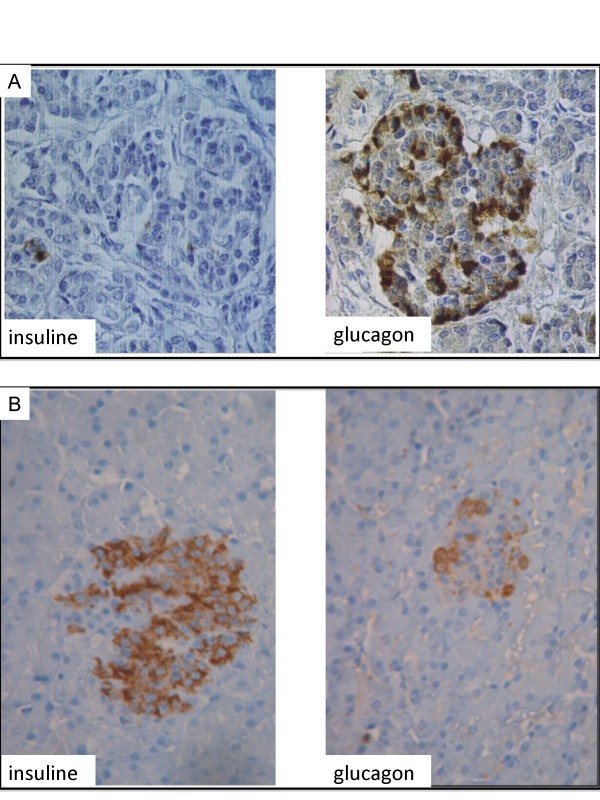
**Pancreatic histology (islets)**. Staining of Langerhans islets using immunoperoxydase with specific antibodies against insulin and glucagon (× 400). A: WRS patient; B: Normal control. In WRS patient, the majority of the cells within the islets stain for glucagon. Numerous cells also stain positively for somatostatin (not shown), and there is a marked reduction in the number of insulin-secreting β cells, which are sparse within the islets. (histological study performed by R. Bouvier and S. Collardeau-Frachon, Lyon).

The pancreas is typically hypoplastic with a reduction of acinar tissue that exhibits evidence of necrosis, with similar histological characteristics to the liver (Figure 6; S. Collardeau-Frachon, pers. communication). Langerhans islets are smaller than normal, in relation with the severe β cell defects, as evidenced by immunohistochemical study showing a major reduction of insulin staining, whereas the majority of cells stain for glucagon (Figure 7) [36].

Overall, both in human WRS and Perk KO mice, the necrotic lesions observed in the liver, kidney and exocrine pancreas appear histologically very similar, contrasting with the absence of similar features in the endocrine pancreas and the bone. Indeed, in β cells and osteoblasts, detailed mouse studies showed clear evidence for proliferation and differentiation defects, rather than cell death [22,24,33]. In view of the broad range of mechanisms controlled by PERK, it is likely that the variety of cellular consequences of PERK deficiency observed in different organs and cell types reflect the variety of the mechanisms and pathways that may be affected, directly or indirectly. The consequences for β cells, resulting in diabetes in WRS patients, appear to affect principally the proliferation and differentiation of the cells, as well as proinsulin trafficking and insulin secretion [33]. Similar mechanisms acting on the highly secreting osteoblasts are likely responsible for the bone defect observed in WRS, as demonstrated in Perk KO mice [24]. In contrast, the necrosis appearance observed in the liver, kidney and exocrine pancreas suggests the implication of distinct mechanisms, timing or regulations, which may result in cell death in a cell-specific manner. Detailed investigation of the exocrine pancreas in exocrine-specific Perk KO mice showed that the mode of cell death in this tissue is oncosis rather than apoptosis, and there was no evidence of defective protein synthesis and secretion, a normal appearance of the ER in these cells, and no evidence of ER stress, but a strong inflammatory response [23]. Finally, recent studies in Perk KO mice suggest that PERK may act as a metabolic sensor in the β cells, to modulate the trafficking and quality control of proinsulin to the physiologic demand in insulin [33]. This adaptive mechanism is likely to play a central role in frequent forms of diabetes and metabolic diseases. The remarkably similar clinical and histological characteristics of human WRS and Perk KO mice stress the value of detailed studies of global and organ-specific Perk KO mice in order to dissect the variety of tissue-specific defects and understand disease mechanisms[22].

## Diagnosis

### Clinical diagnosis

WRS should be suspected in any infant who presents with permanent neonatal diabetes associated with skeletal dysplasia and/or episodes of acute liver failure. Since part of the key presenting features of the syndrome may occur later during the course of the disease, WRS should be suspected in any infant with diabetes mellitus occurring neonatally or at a very young age (before 6 months) originating from a population where there is a high prevalence of consanguinity, or in case of history of neonatal diabetes with rapidly fatal outcome in siblings or in the extended family; these patients should be assessed radiologically for early signs of skeletal abnormalities and deficient mineralization. Growth retardation and short stature in WRS patients can generally be documented clinically only after the age of one year or so. From that age, gradual slowing of growth rate is generally observed with growth retardation that sometimes becomes extremely severe. Growth parameters are progressively below 5 SD for the age with time (Figure 2B). For older patients, there is a tendency for a permanent cessation of growth after the age of 10 years, in relation with the development of bone lesions. It should be noted that the extent of the clinical variability of the syndrome may not be fully known at present, as a consequence of its rarity, and age at onset, bone dysplasia and liver failure should not be required for suspecting WRS in a patient with early-onset diabetes. Homozygous *EIF2AK3 *mutations have been found in WRS patients with age at onset up to 2.5 years [2], in one WRS patient with no bone dysplasia at age 32 years [4], and hepatic failure has not been reported in all WRS patients [3]. Although WRS is a rare syndrome, it now appears to be the most frequent form of neonatal/early-onset permanent neonatal diabetes in patients born from consanguineous parents, where it may account for 25% of cases [4]. We suspect that WRS is largely underdiagnosed at present because of the early death of patients and/or the higher prevalence of these patients in countries where structures for health care may not be optimal.

### Genetic diagnosis

In view of the relatively high prevalence of WRS syndrome evidenced by *EIF2AK3 *mutations in neonatal/early onset diabetic patients born from consanguineous parents, we recommend systematic sequencing of the coding regions of *EIF2AK3 *gene in patients born from consanguineous parents, or coming from isolated populations with frequent inbreeding, even in the absence of any evidence of osteoporosis or bone abnormalities at disease onset. This early diagnosis is important in order to follow up these patients more carefully, and in particular to insure rapid care during episodes of hepatic failure.

## Differential diagnosis

WRS should be differentiated from other forms of neonatal or early-onset insulin-dependent diabetes, based on the clinical presentation. Ultimately, *EIF2AK3 *mutation screening confirms (or excludes) the WRS diagnosis. The hypothesis of a variant form of WRS, not caused by *EIF2AK3 *mutation and with variant clinical features, remains a possibility, but should be confirmed in additional cases [2].

WRS is always permanent after disease onset, which easily distinguishes it from transient neonatal diabetes, which is caused by other genetic defects. Most cases of PNDM occurring before the age of 6 months are thought to be largely genetically distinct from typical T1D, based on the absence of distortion of HLA class II allele distribution compared to control individuals [37,38]. In contrast to T1D, WRS patients do not have β cell specific autoantibody at disease onset.

Other genetic causes of PNDM have been identified, in which diabetes may be isolated (non-syndromic) or syndromic. Non-syndromic diabetes can be caused by dominant or recessive mutations in the potassium channel genes *KCNJ11 *and *ABCC8*, or the insulin gene (*INS*), or by recessive mutations in glucokinase gene (*GCK*); additional neuro-motor and neuro-psychological abnormalities are frequently observed in patients with *KCNJ11 *and *ABCC8 *mutations. In outbred Caucasian populations, heterozygous mutations in *KCNJ11*, *ABCC8 *or *INS *genes may account for almost 40% of PNDM, compared to less than 5% in consanguineous families; in contrast homozygous mutations at *INS*, *GCK *or *ABCC8 *genes may account for a total of 30% and *EIF2AK3 *mutations for almost 25% of PNDM patients in consanguineous families [4]. Although WRS is easily distinguished from these other forms of PNDM based of the additional clinical features, they may be mistaken at the time of diabetes diagnosis, when the rest of the syndrome is not yet expressed. Careful inspection of early signs of deficient bone mineralization and skeletal dysplasia should be searched for at the time of diagnosis. Although the age at onset of diabetes and birth weight may be additional distinctive features, in that patients with *EIF2AK3 *and *ABCC8 *homozygous mutations tend to have a later age at onset and slightly greater birth weight than patients with homozygous mutations at *GCK *and *INS *[4], this criteria may not be fully diagnostic because of the overlap of distributions.

Other rare autosomal recessive forms of PNDM, which are associated with various organ impairments, have been genetically characterized and are easily differentiated from WRS: *PDX1 *mutations cause pancreas agenesis with additional exocrine pancreas deficiency [39], or a variant form with only neonatal diabetes [40]; *PTF1A *mutations cause pancreas and cerebellar agenesis [41]; *GLIS3 *mutations cause neonatal diabetes, congenital hypothyroidism and other clinical features [42]. *FOXP3 *mutations (X-linked) are responsible for neonatal diabetes syndrome associated with X-linked immune dysregulation and enteropathy [43].

The short stature related to skeletal dysplasia, regardless of other dysfunctions that occur in patients with WRS, may be evocative of other spondylo-epiphyseal dysplasias such as mucopolysaccharidoses. Early-onset T1D occurring in these patients may also lead to a similar phenotype as WRS. In such cases however, disease onset is likely to be older, and β cell specific autoantibodies should differentiate them from true WRS.

In summary, in view of the straightforward genetic diagnosis, we recommend *EIF2AK3 *mutation screening for diagnosis in all neonatal/early-onset diabetes patients (at least before age of 6 months) with clinical features specific for WRS, or in the absence of extra-pancreatic manifestations in PNDM patients born from consanguineous parents.

## Genetic counselling and antenatal diagnosis

Genetic counselling of WRS is strongly recommended. As a rare autosomal recessive disease, with no clinical manifestations in parents, genetic counselling can only be proposed to parents of a previous diagnosed WRS child, with confirmed *EIF2AK3 *mutation. Recurrence risk is 25% in siblings of WRS patients. In cases of extended consanguineous families with a genetically diagnosed WRS case, other related consanguineous couples should also benefit from genetic counselling, and their carrier status for the *EIF2AK3 *mutation determined.

In view of the early disease onset and severe condition and prognosis of WRS at present, prenatal diagnosis is strongly recommended for this disease. Pregnancies are generally uncomplicated, and the only prenatal diagnosis of WRS is genetic diagnosis, based on genotyping the *EIF2AK3 *mutation(s) that is(are) present in both parents (heterozygous carriers).

## Clinical management

In terms of diabetes, close therapeutic monitoring should be recommended because of the tendency for frequent acute episodes of both hypoglycaemia and ketoacidosis. Under this context, treatment with insulin pump is recommended, especially in small infants, in order to optimize the prevention of severe hypoglycaemia. Generally, in contrast with patients affected with other forms of diabetes, treatment with insulin should not target a very tight control of blood glucose, in order to avoid hypoglycemia, which may trigger episodes of acute aggravation of the disease. In practice, children with WRS have a risk of developing acute multi-organ failure during intercurrent illness. It is very important to inform the patient's relatives in order that they can recognize these episodes very early so that hospitalization can be organized very rapidly and appropriate symptomatic treatment instituted. Bone fractures may be frequent and must be managed at the orthopaedic level. In WRS patients, interventions under general anaesthesia increase the risk of acute aggravation, as a consequence of the toxicity of anaesthetics, and should be avoided when possible. Similarly, any drug or vaccine not strictly necessary should be limited, taking into account the risk of triggering secondary liver and/or kidney failure.

## Prognosis

The prognosis is poor, and WRS patients generally die at a young age. In a review of 19 patients with known age at death, only 3 died at 10 years or older [3]. Two patients were reported with a longer survival, until 35 years for one patient [2], and alive at 32 years old for one patient [4]. In general, in the final episode of aggravation, the patient is initially admitted to the hospital with flu-like symptoms and fever. Death occurs later in a situation of multi-organ failure with predominant liver and renal dysfunction, hepatic failure being sometimes associated with encephalopathy. Remarkably, acute liver episodes had not been reported in the patient who survived until age 35 years.

## Future prospects

In view of the observed ER dysfunction in WRS patients, intervention strategies targeting a reduction of ER stress or other pathways involved in this dysfunction are of potential interest for therapy and possibly prevention. Recent studies targeting ER stress reduction using chemical chaperones, glucagon-like peptide-1 (GLP-1) agonists or other strategies have reported promising results *in vitro *and *in vivo *(cellular and animal models), and in particular regarding protection of β cells from ER stress [31,44]. Wolfram syndrome (WFS), a monogenic diabetes, is caused by mutations in the WFS1 gene, another important ER gene. Interestingly, treatment of *Wfs1 *KO mice with pioglitazone was found to reduce β cell loss and protect mice from diabetes [45]. As ER stress related mechanisms, or possibly other ER disturbance, have been suggested not only for monogenic diabetes such as WRS and WFS but also in frequent forms of diabetes, such as T1D and T2D, and in other common conditions including obesity and cardiovascular diseases, such intervention strategies may have very large applications for health purpose.

## List of abbreviations

WRS: Wolcott-Rallison syndrome; EIF2AK3: eukaryotic translation initiation factor 2α kinase 3; ER: Endoplasmic Reticulum; PERK: PKR (double-stranded-RNA-dependent protein kinase)-like ER kinase; UPR: Unfolded Protein Response; GAD: Glutamic Acid Decarboxylase; IA2: Tyrosine Phosphatase; ICA: Islet cell antibody; PNDM: Permanent Neonatal Diabetes Mellitus; CT: Computed Tomography; MRI: Magnetic Resonance Imagining; T1D: Type 1 Diabetes; T2D: Type 2 Diabetes; WFS: Wolfram Syndrome; SD: Standard Deviation; HLA: Human Leucocyte Antigen.

## Competing interests

The authors declare that they have no competing interests.

## Authors' contributions

The authors contributed equally to this review. They read and approved the final version of the manuscript.
